# Folded flexure MOEMS for the detection of PSA and hepatitis DNA as biosensor for prostate cancer and viruses

**DOI:** 10.1038/s41598-024-73910-x

**Published:** 2024-10-02

**Authors:** Hossein Bahramian, Jalal Gholinejad, Arash Yazdanpanah Goharrizi

**Affiliations:** Department of Electronics, Faculty of Electrical Engineering, Shahid Beheshti University (SBU), Evin, Tehran, 19839- 69411 Iran

**Keywords:** Biosensor, Prostate specific antigen (PSA), Hepatitis DNA, MOEMS, Folded-flexure, Applied optics, Optical materials and structures, Optical techniques

## Abstract

Micro-opto-electro-mechanical systems (MOEMS) biosensors are employed in various applications such as disease monitoring, drug investigation, detection of pollutants, and biological fluid studies. In this paper, a novel MOEMS biosensor based on a differential folded-flexure structure is introduced. The designed device is employed to detect prostate-specific antigen (PSA) protein and Hepatitis DNA. The target molecules cause a mechanical deflection in the folded-flexure; subsequently, the transmitted optical power across the finger, attached to the flexure, is modulated in proportion to the input concentration. Then, a photodiode power sensor measures the modulated optical power, where the output of the sensor is simply a current related to the target molecules’ concentrations. The employed readout circuit operates at a wavelength of λ = 1550 nm with a laser power of 1 µW. The dimensions of the proposed biosensor are considered to be 365 × 340 × 2 μm³, making this sensor small enough and suitable for integration. The designed biosensor provides notable features of mechanical deflection sensitivities of 0.2053 nm/(ng/ml) and 7.2486 nm/nM, optical transmittance sensitivities of 0.535504 × 10^−3^ 1/(ng/ml) and 18.91 × 10^−3^ 1/nM, total output sensitivities of 0.5398 (mA/W)/(ng/ml) and 19.059 (mA/W)/nM, and measurement ranges of 0-1000 ng/ml and 0-28.33 nM for PSA and Hepatitis DNA, respectively. The proposed system is a sensitive and powerful sensor that can play an important role in diagnosing many diseases.

**Supplementary Information:**

The online version contains supplementary material available at 10.1038/s41598-024-73910-x.

## Introduction

Nowadays, Micro-opto-electro-mechanical systems (MOEMS) sensors have essential role in several fields, including medicine, DNA sequence detection, toxin detection, blood pressure control, and etc^1–4^. MOEMS biosensors operate based on biological principles, and are employed for diagnosis of diseases^5–8^. The environment in which MOEMS biosensors operate can include human body, plant, water, air, soil, and biological habitat^9–13^.

Easy use, low cost, and reliability are of characteristics of these sensors, and it is important to develop new methods for designing of MOEMS biosensors^14–16^. Based on this, the field of MOEMS biosensors is known as one of the main fields of medical engineering currently. Moreover, the use of biosensors in various fields such as medicine, biology, agriculture and environment is increasing as one of the effective methods to progress and improve people’s living conditions. For instance, biosensors are used to detect DNA sections of human^17^. However, there is a significant need for novelty in optical readout circuits (ROCs)^18^.

Besides, MOEMS biosensors are hired to detect molecules that demonstrate the risk of cancer in human. For instant, detection of PSA can help for diagnosis of prostate cancer, and it is used as a tumor marker^19^. PSA is a protein that is primarily made by prostate cells, and is a glycoprotein also known as kallikrein 3 (KLK3)^20^. In fact, a significant increase in PSA in the blood may be associated with prostate cancer^21^.

MEMS cantilevers are employed as suspended nanomechanical resonators for DNA weighing which is used to detect virus^22^. The designed sensor was sensitive to zeptogram range, and just microliters of sample were needed for this device. Moreover, nanomechanical array has been hired to detect SARS-CoV-2 variants^23^ and to analyze malaria vaccine^24^. Here, a single-step evaluation via piezo-actuated microcantilevers is described.

Larry O’Connell^25^ reported the ability of a cantilever array biosensor in dynamic mode for detection of oligonucleotides. This study utilized gold nanoparticles; however, the system’s noise level was unfavorable, indicating a potential requirement for differential measurements in ROC analysis. Timothy A. Okhai et al.^26^ introduced an electrochemical device employing anti-PSA antibody (Ab) and silver nanoparticles (AgNPs). The device demonstrated a detection range of 2.5 to 11.0 ng/mL with high linearity.

Naresh Mandal et al.^27^ introduced a modified glassy carbon electrode that was modified via silver nanoparticles for the detection of PSA. The response range of the designed biosensor was 1 pg/ml to 3 µg/ml. Dong Gun Hwang et al.^28^ presented a bridge-shaped resonator based on PZT for recognition of PSA. A concentration range of 10 pg/ml to 100 ng/ml was obtained with small dimensions of the biosensor.

Min Yue et al.^29^ proposed a label-free MOEMS cantilever for detection of Hepatitis virus detection. In this work, optical interferometry method was utilized. Min Yue et al.^30^ introduced a 2-D micro-cantilever array for multi-purpose bio-detection procedure. Here, the immobilization of thiolated single-stranded DNA (ssDNA) was investigated, and the ROC was based on laser ray detection.

In the present work, we introduce an innovative structure, capable of detecting both PSA and Hepatitis virus. The designed biosensor is based on a folded-flexure with a differential measurement method, and an optical technique is employed in the ROC analysis. The mentioned targets induce a mechanical deflection on the designed spring, resulting in a change in the transmitted optical power proportional to the targets’ concentration. The results demonstrate high sensitivity, wide detection range, and integrable dimensions.

## Design procedure

In this section, we present the employed structure, followed by an explanation of the measurement technique and the proposed parameters of the device.

### Folded-flexure structure

**Fig. 1:**
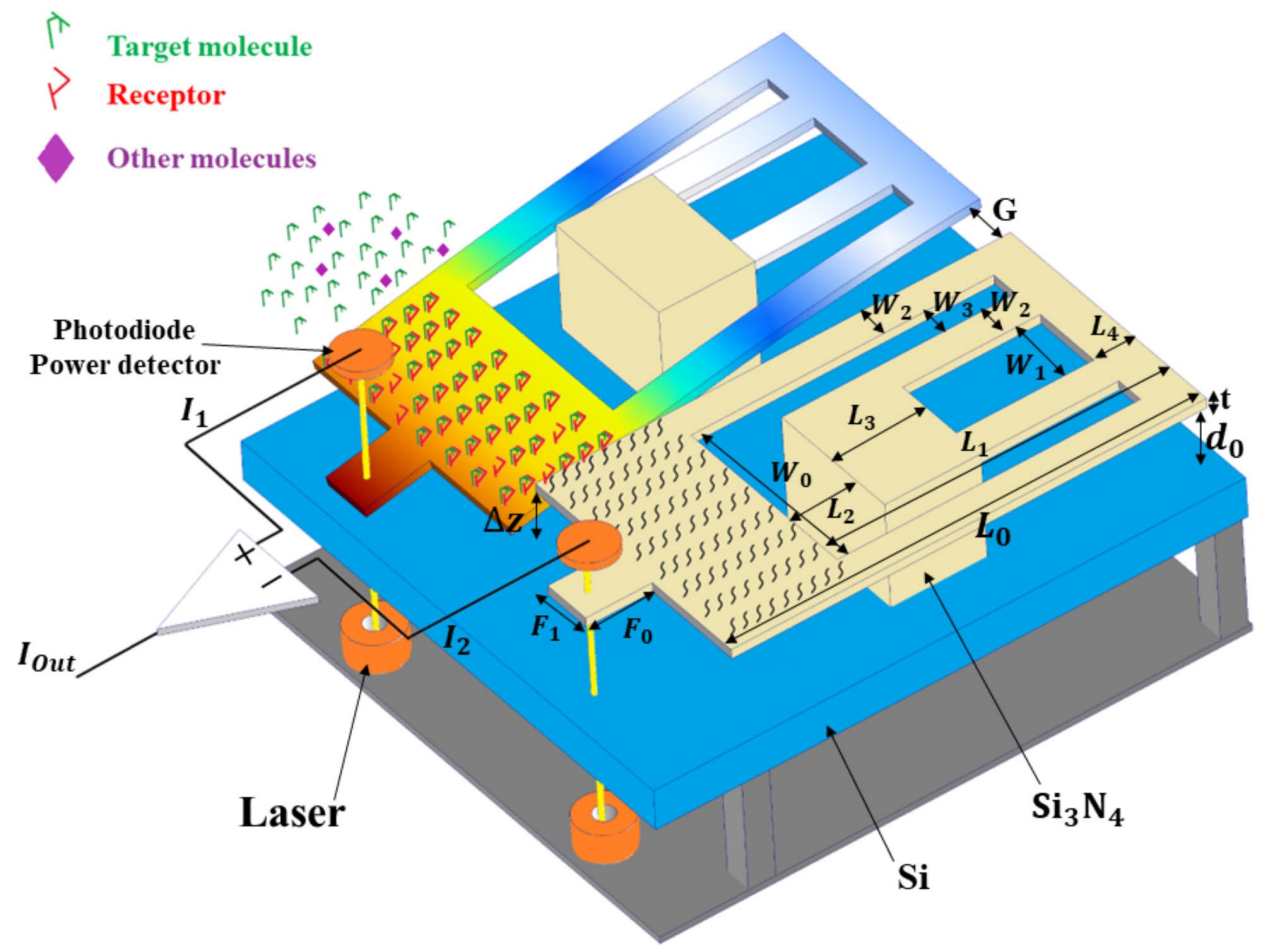
A general view of the packing and elements of the designed biosensor. The biosensor contains two folded flexures, laser, and photodiode. One flexure is covered with receptors which are sensitive to the target molecules. The other flexure is free by the purpose of differential measurement. When the target molecules enter to the structure, a deflection is induced on the sensing flexure. Via target molecule’s concentration, the distance between finger and substrate is modulated. Subsequently, the transmitted optical power is changed in accordance to the target molecule’s concentration.

Figure 1 illustrates the schematic of the sensor, depicting the flow of biological fluid into a Si_3_N_4_folded-flexure structure. On one of the folded-flexures, the target molecule’s receptor is layered (target flexure), and the other flexure is clear (free flexure) with the purpose of differential measurement. In fact, the target molecules interact with flexure, covered with receptors, and are banned from free flexure. Other molecules produce distortions, and create an equal mechanical movement on both flexures. When the target molecule enters, only one spring is bent according to its concentration^19,29^, and this bending changes the air gap between the ROC finger and the substrate. As a result, the amount of transmitted light that reaches the photo-detector (PD) (S132C) is modified. Finally, at the output we have two currents: one corresponds to the changes of target molecule concentration plus the other molecules’ noise, and the second current is due to only the noise caused by other molecules. By the difference of the mentioned currents, the output signal is due to the concentration of the target molecule, and the noise signal is removed from the total output of device. To simulate the introduced approach, numerical studies are employed, where the mechanical deviation caused by the target molecules are simulated using the force applied to the proof mass via COMSOL. Then, the amount of displacement of the proof mass is obtained, and it is used for finger movement in LUMERICAL FDTD. The designed measurement approach is simple, and can provide a continuous monitoring. Moreover, “GIF S1-Folded MOEMS Biosensor” show the mentioned measurement procedure, and the mechanical and optical parameters of the designed biosensor are listed in Table 1.

**Table 1:** The parameters of the designed biosensor.

Parameter	Explain	Value
$\:{L}_{0},\:{L}_{1},\:{L}_{2},\:{L}_{3},$ and $\:{L}_{4}$	Length dimensions	350, 250, 50, 70, and 30 μm
$\:{W}_{0},\:{W}_{1},\:{W}_{2},\:{W}_{3},$ and $\:{W}_{4}$	Width dimensions	112, 52, 24, 6, and 24 μm
$\:{F}_{0}$ and $\:{F}_{1}$	Sensing finger size	15 and 5 μm
$\:{d}_{0}$	Substrate distance	0.34 μm
t	Thickness	0.5 μm
G	Two flexure distance	20 μm
E	Si_3_N_4_ Young’s modulus	80 GPa
n	Si_3_N_4_ Poisson’s ratio	0.24
*C*	Concentration ranges	0-1000 ng/ml (PSA) 0–28. 33 nM (Hepatitis DNA)
R [31]	PD responsivity	1.0081 A/W
P	Laser power	1 µW
λ	Wavelength	1550 nm

### Sensing technique

**Fig. 2:**
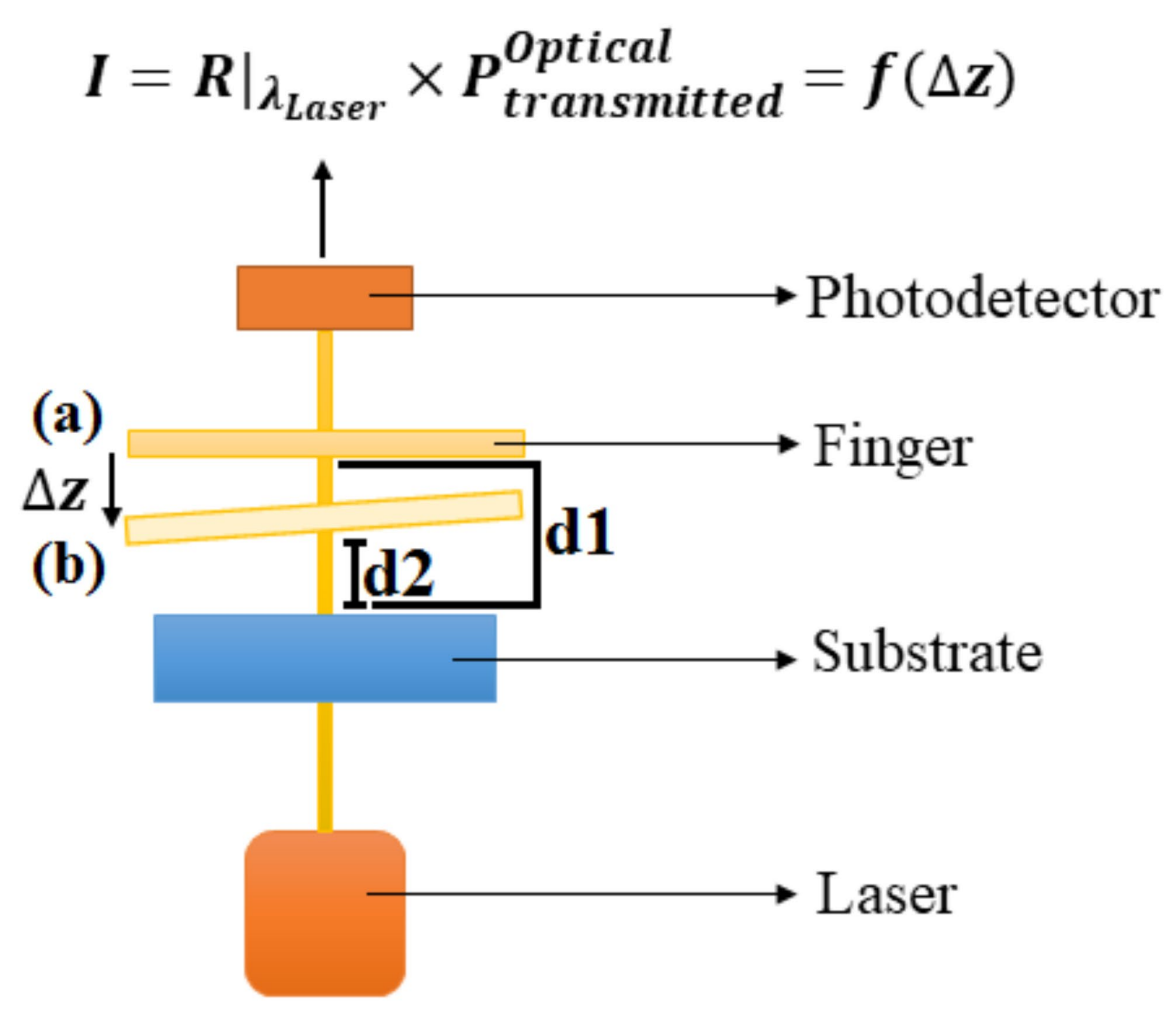
The procedure of optical sensing. As shown, the position of the finger is changed according to the concentration of target molecules, (**a**) before and (**b**) after adsorption of target molecules.

In Fig. 2, the cross-section view of the optical ROC is displayed. As seen, the measurement starts with a mechanical tilt (∆z = d1-d2) of folded-flexure due to the molecules’ concentrations. Subsequently, the transmitted optical power is modulated, and the output currents are achieved. The output current is changed based on ∆z which is associated with the target molecules’ concentration.

To discuss the operational mechanism of the proposed biosensor, the modeling of both target and free flexures (with receptors and without receptors) is described as follows. The model of the target flexure can be explicated in terms of the concentration of the target (e.g., PSA or Hepatitis DNA) and background noises (unwanted molecules, fabrication defects, or deformation problems due to intrinsic stress issues):

1 $$\:{C}^{target}+{C}^{noise}\to\:{\varDelta\:z}_{1}\to\:{T}_{1}\to\:{I}_{1}$$

where $\:{C}^{target}$ is the concentration of the target molecule, $\:{C}^{noise}$ is due to other molecules, $\:\varDelta\:z$ is the mechanical deflection of the folded-flexure, $\:T$ is the transmittance of optical ROC, and $\:I$ is the output current of PD. As well, the model of the free flexure is:

2 $$\:{C}^{noise}\to\:{\varDelta\:z}_{2}\to\:{T}_{2}\to\:{I}_{2}$$

This means that the free flexure is not affected by target molecules, and its movements are due to other substances. Subsequently, the output current from target flexure can be calculated as:

3 $$\:{I}_{1}=RP{T}_{1}\sim{\varDelta\:z}_{1}\sim({C}^{target}+{C}^{noise})$$

where $\:R$ is the responsivity of PD and $\:P$ is the optical power of laser. As the same for free flexure, it can be written:

4 $$\:{I}_{2}=RP{T}_{2}\sim{\varDelta\:z}_{2}\sim{C}^{noise}$$

Consequently, the output signal of the device is as follows:

5 $$\:{I}_{out}=\varDelta\:I={I}_{1}-{I}_{2}\sim{C}^{target}$$

**Fig. 3:**
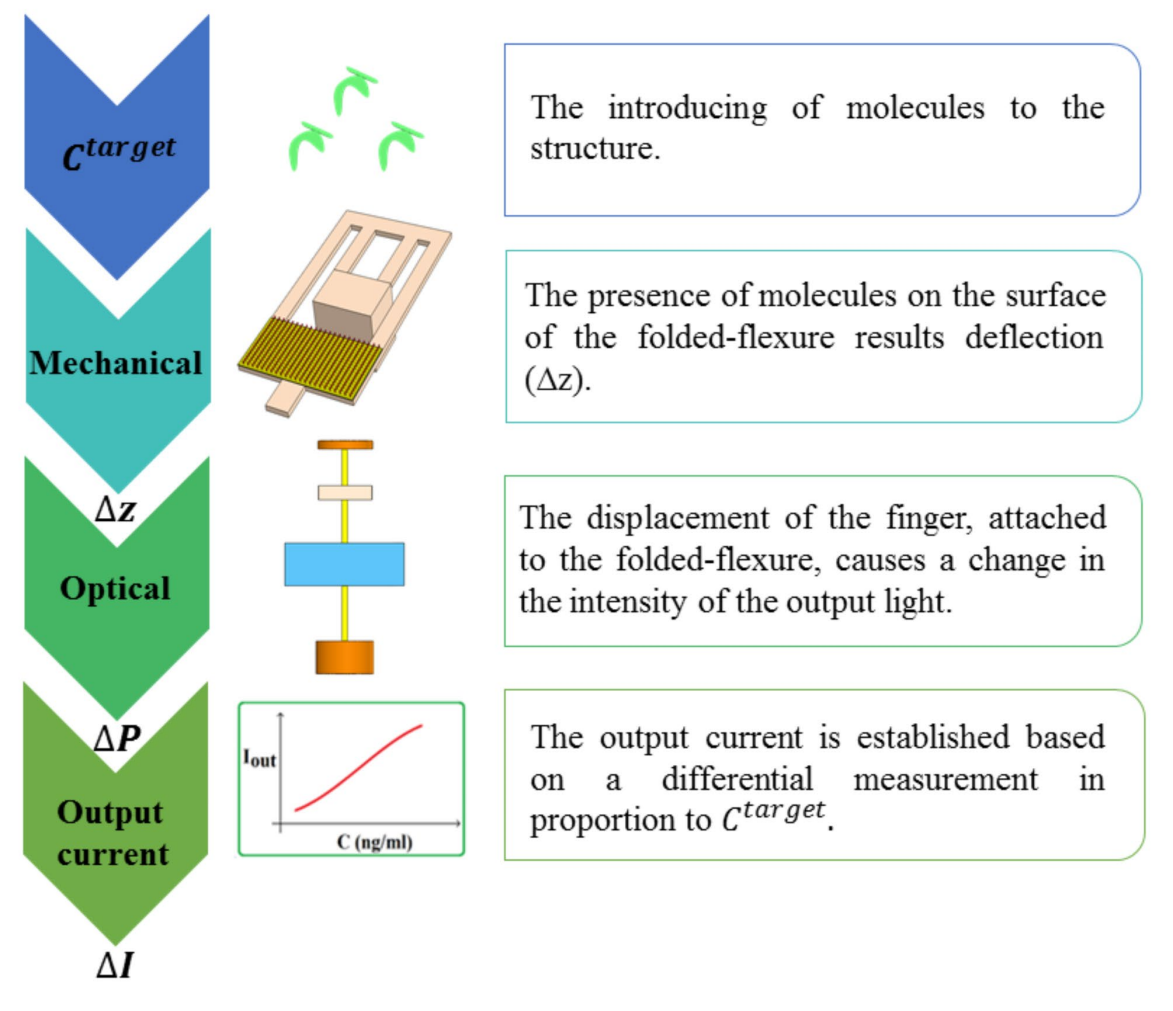
The illustration of the sensing approach. The target molecules enter into the mechanical structure, and proportional to their concentration a deflection is induced within the folded-flexure. Subsequently, this deflection alters the air gap between the finger and the substrate in the optical mechanism. This results a change in the intensity of the transmitted light, ultimately the current of PD is modulated.

As seen in Fig. 3, the algorithm and the overview of sensing manner is illustrated. While the employed technique is simple, it is established based on an effective method. The input concentration modulates the movement of mechanical structure. Afterwards, the distance of finger in the optical ROC is changed, and the transmitted optical power is varied. Finally, the output current is obtained.

### Suggested fabrication process

**Fig. 4:**
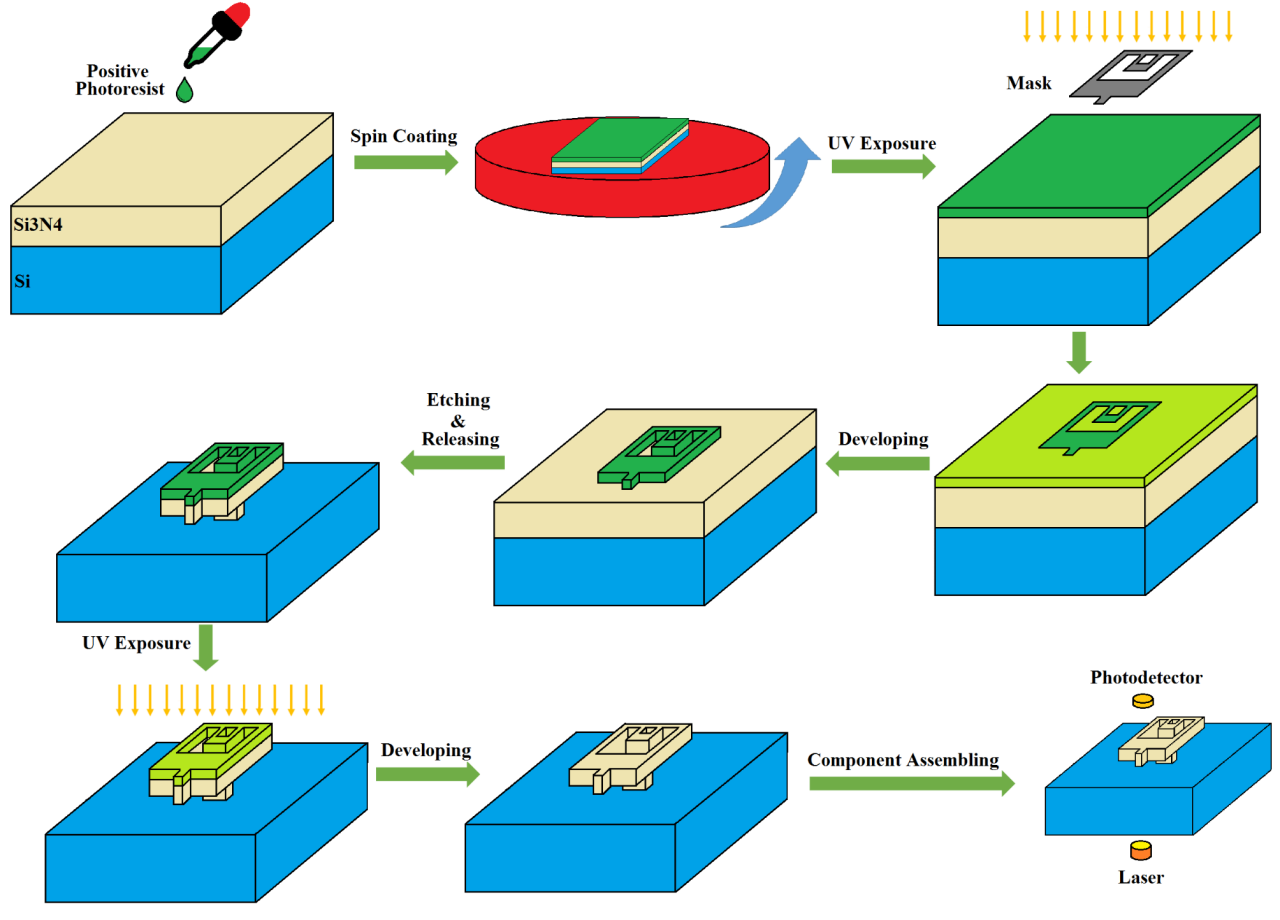
The illustration of the suggested fabrication process. The procedure starts with a prepared Si-Si_3_N_4_ wafer, where a positive photoresist is layered on it via spin coating. Subsequently, the stages of UV exposure under mask, developing, and etching are employed to achieve folded-flexure structure. Finally, the PD and laser are assembled to the sensor.

The suggested fabrication process is proposed in Fig. 4, where the procedure employs common photolithography functions. It starts with the spin coating of a positive photoresist on a prepared wafer, afterwards using an appropriate mask, UV exposure is done. Next, developing and wet etching steps are used to produce the folded-flexure structure. Finally, the laser and PD are aligned, and the calibration can be done.

## Result and discussion

In this section, the mechanical and optical simulation results are provided, where for simulations COMSOL and LUMERICAL FDTD softwares are utilized. Moreover, a comparative study is done.

### Mechanical study

**Fig. 5:**
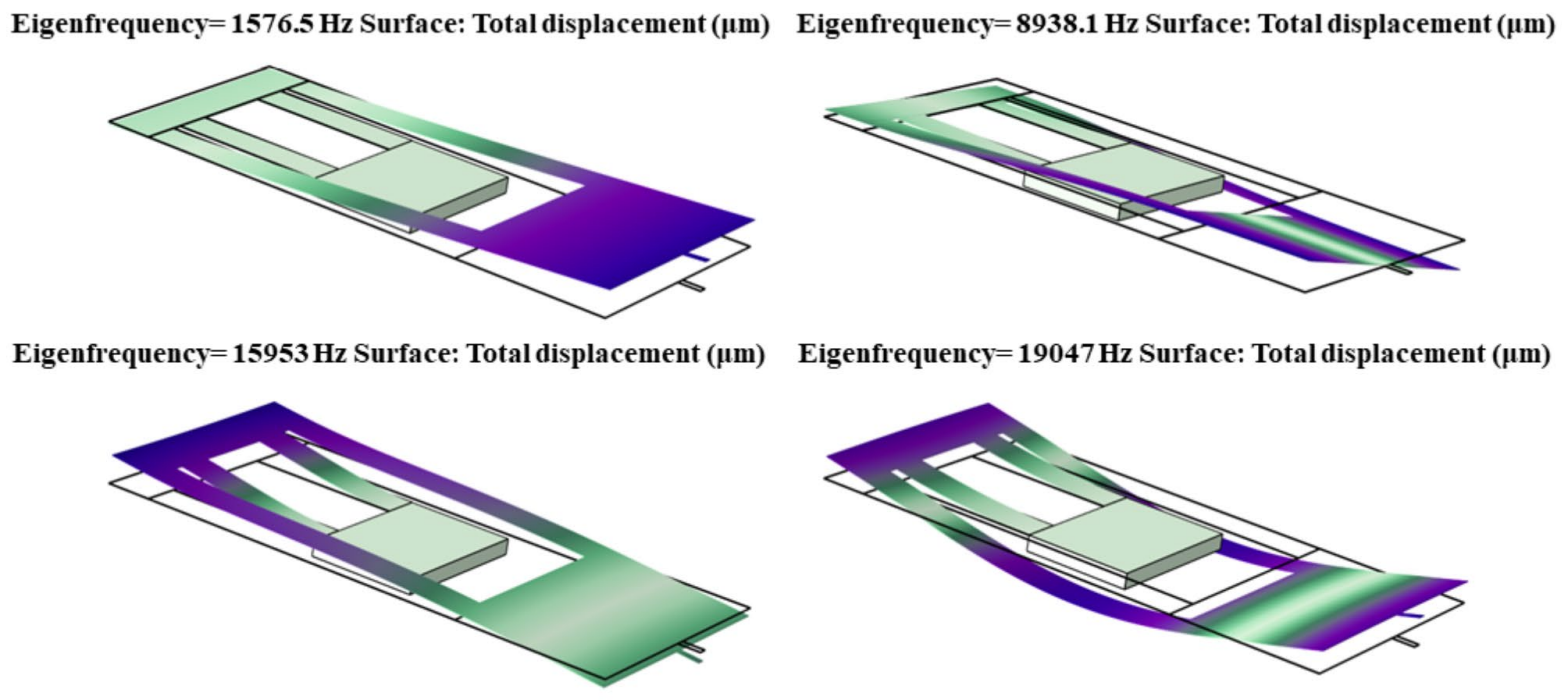
The first four frequencies of the structure are determined to be 1.57, 8.93, 15.95, and 19.04 kHz, respectively. These frequencies exhibit significant deviation from natural noise frequencies, and indicate a structurally stable configuration.

**Fig. 6:**
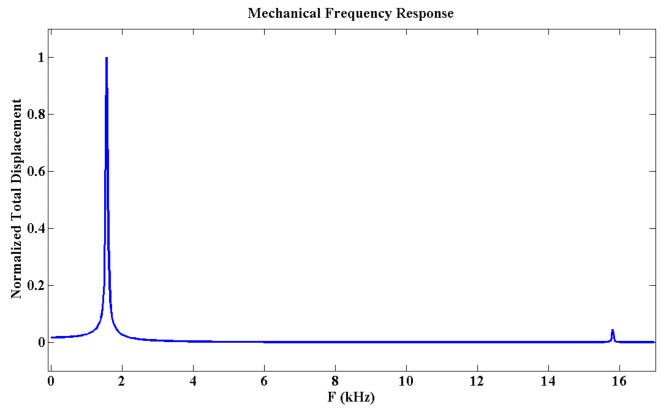
The frequency response of the designed folded flexure. The first resonance frequency (1.57 kHz) is dominant.

To assess the stability of the proposed biosensor, the frequency response of the MOEMS folded-flexure is calculated and is illustrated in Fig. 5. As depicted, the Eigen frequencies of the utilized folded-flexure in the MOEMS biosensor are determined to be 1.57, 8.93, 15.95, and 19.04 kHz. The obtained mechanical frequencies of the proposed structure are significantly different from the resonance frequencies of natural noises. According to the simulation results of this study, among these mechanical frequencies, the frequency of 1.57 kHz emerges as the dominant frequency of the structure, as shown in Fig. 6.

**Fig. 7:**
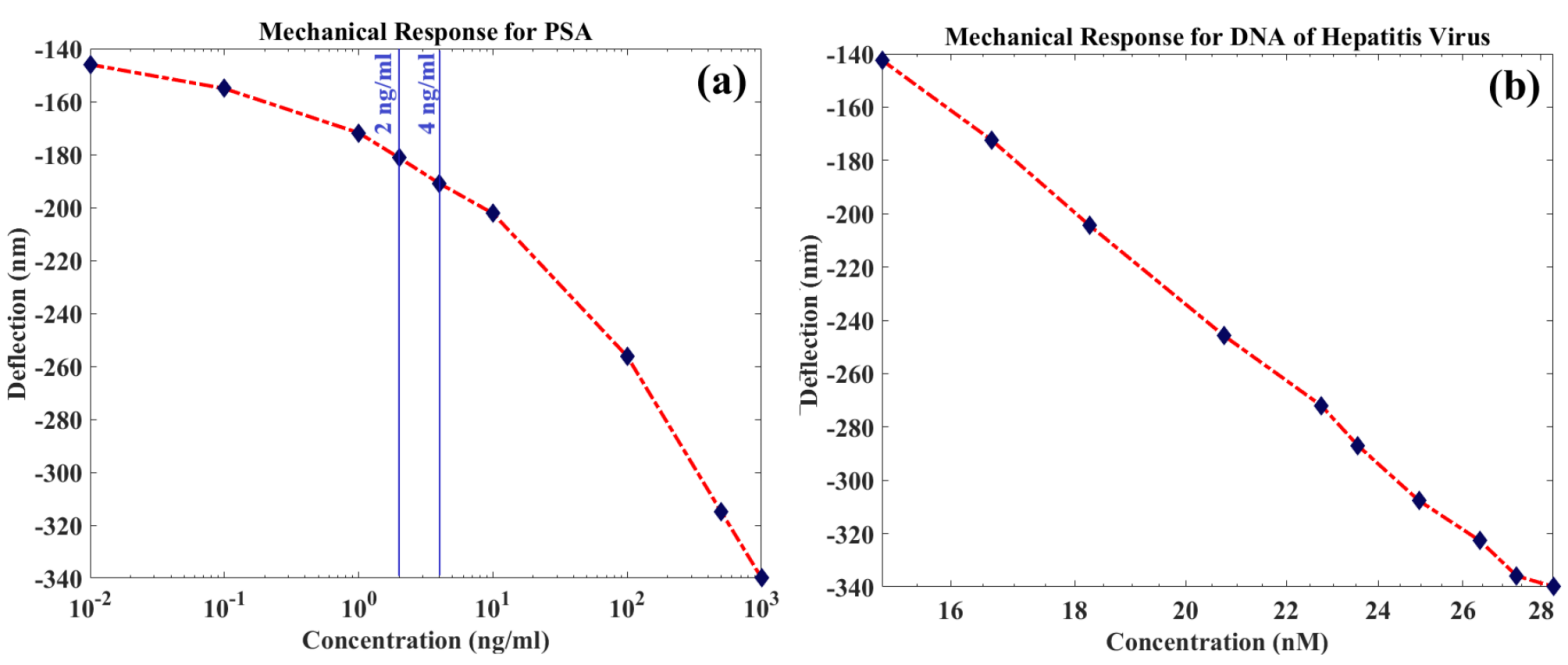
Structure displacement (∆z) as a function of concentration for (**a**) PSA and (**b**) Hepatitis DNA. For both targets, wide input ranges are obtained by the structure.

By applying various concentrations of PSA and Hepatitis DNA the mechanical deflection of the folded-flexure structure (∆z) is achieved in Fig. 7. The clinical critical level for PSA concentration in different references is reported as 2 ng/ml^19^ and 4 ng/ml^20,32^. As the simulation results are shown in Fig. 7a, in the proposed biosensor structure, the folded-flexure undergoes significant deflection in response to the mentioned concentration of PSA molecules, demonstrating the sensor’s efficiency. If the concentration of the PSA is greater than its clinical critical level in the sample, the amount of bending is increased. According to Fig. 7(a), a mechanical sensitivity of 0.2053 nm/(ng/ml) for range of 0-1000 ng/ml of PSA is acquired. In Fig. 7b the response of the proposed biosensor for the diagnosis of Hepatitis is illustrated. As the figure shows, by increasing Hepatitis DNA concentration the deflection is increased almost linearly. Based on the results a mechanical sensitivity of 7.2486 nm/nM for 0-28.33 nM of Hepatitis DNA is accomplished.

### Optical results

**Fig. 8:**
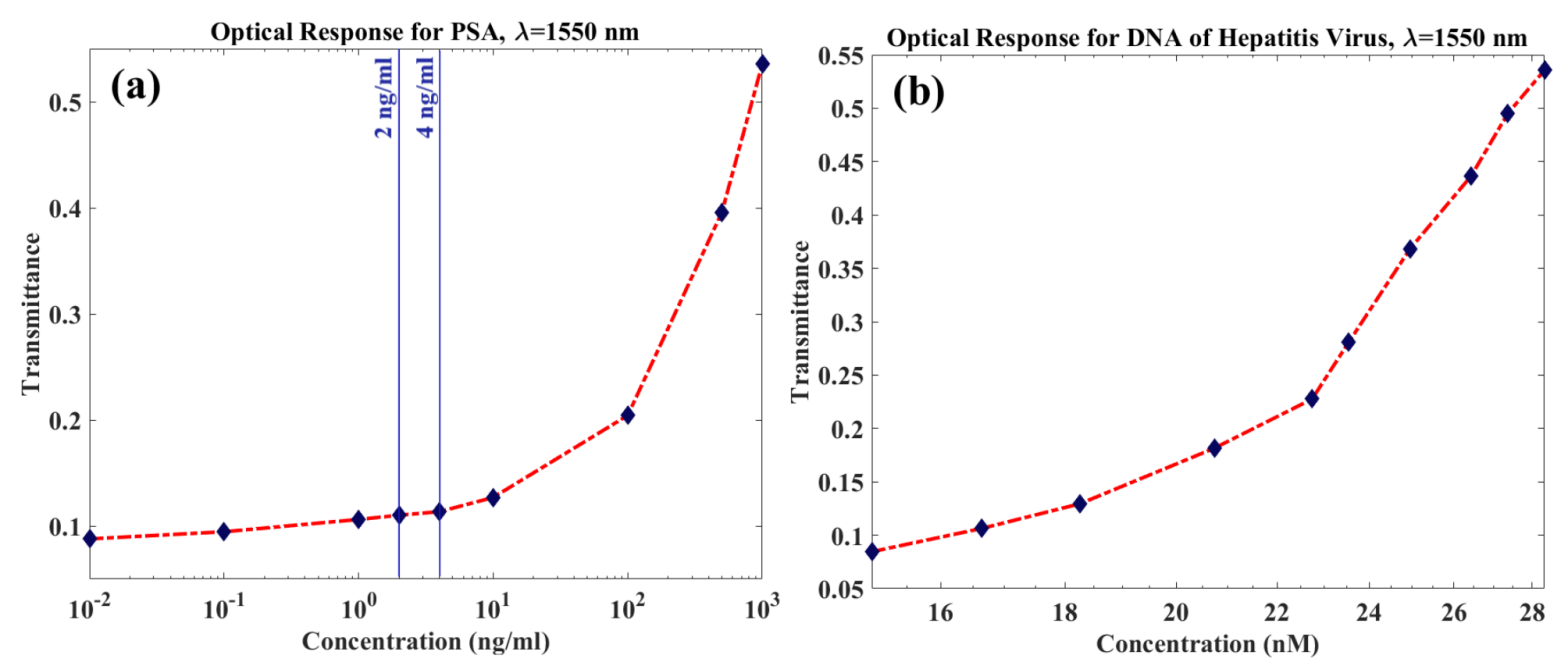
The transmittance response (T) at λ = 1550 nm for (**a**) PSA and (**b**) Hepatitis DNA against their various concentrations.

The mentioned mechanical deflections (∆z) of both target molecules are used to simulate the transmittance response of ROC. As seen in Fig. 8, by changing the gap between the finger of the flexure and the substrate due to the altering of concentration, the transmittance is increased. This means that the transmitted optical power is modulated in proportion to the target concentration. Therefore, transmittance sensitivities of 0.535504 × 10^−3^ 1/(ng/ml) and 18.91 × 10^−3^ 1/nM are obtained for PSA and Hepatitis DNA, respectively.

**Fig. 9:**
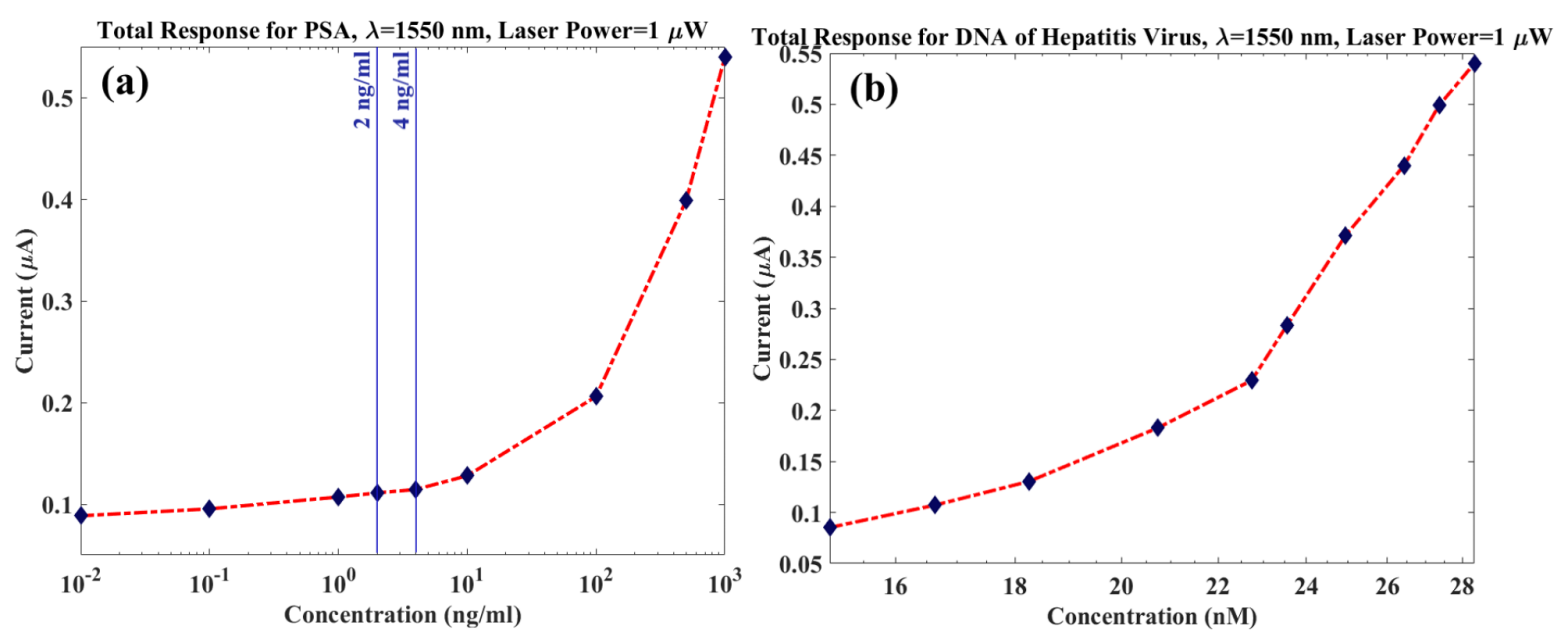
The output current of sensor as a fuction of concentration for (**a**) PSA and (**b**) Hepatitis DNA for 1 µW of laser power at λ = 1550 nm.

By considering an optical laser power of 1 µW and responsivity of S132C (Photo Diode Power Sensor)^31^, the output current of PD is calculated in Fig. 9. This curve is achieved based on Eq. (5), and the employed wavelength is set to be λ = 1550 nm that is commercial in photonics. To observe higher currents in the output, more optical power from laser should be employed. As seen, a broad input concentration range with a significant response is provided via the designed structure with a simple and effective sensing approach. Consequently, total sensitivities of 0.5398 (mA/W)/(ng/ml) and 19.059 (mA/W)/nM are attained for PSA and Hepatitis DNA, respectively.

### Comparative survey

Table 2 compares the parameters of the designed biosensor with previous reported works. The employed wavelength is set to be in communication range which is appropriate for fiber optics. Besides, the obtained dimensions are integrable without need for array measurement technique. Moreover, input concentration ranges are wide. Furthermore, the designed biosensor is capable to be employed for detection of various substances.

**Table 2:** The functional parameters of the designed biosensor in comparison with previous works.

	Mechanical Sensitivity	Optical Sensitivity	Total Sensitivity	Operational Wavelength	Size	Concentration Range	Target
Folded MOEMS (This work)	0.2053 nm/(ng/ml)	0.535504 × 10^−3^ 1/(ng/ml)	0.5398 (mA/W)/(ng/ml)	1550 nm	365 × 340 × 2 µm^3^	0-10^3^ ng/ml	PSA
Microcantilever array [19]	0.111 (mJ/m^2^)/(ng/ml)	-	-	632.8 nm	2.5 cm in diameter	0-10^5^ ng/ml	PSA
CV AgNPs [26]	-	-	0.29 A/(ng/ml)	650 nm	6 × 4 × 4 µm^3^	2.5–11 ng/ml	PSA
Folded MOEMS (This work)	7.2486 nm/nM	18.91 × 10^−3^ 1/nM	19.059 (mA/W)/nM	1550 nm	365 × 340 × 2 µm^3^	0-28.33 nM	Hepatitis
Micro-cantilever array [33]	-	30.21 ppm/(ng/ml)	-	-	10 × 10 mm^2^	0.04–100 ng/ml	Hepatitis
Ni cantilever [34]	-	-	-	633 nm	-	0.04–100 ng/ml	Hepatitis

## Conclusion

MOEMS biosensors are important for detection of various diseases. Here, a MOEMS biosensor using a folded-flexure structure and an optical readout circuit is designed to measure the concentration of both PSA protein and Hepatitis DNA. The introduced device has a size of 365 × 340 × 2 µm^3^, and operates at wavelength of λ = 1550 nm. Moreover, significant characteristic of mechanical deflection sensitivities of 0.2053 nm/(ng/ml) and 7.2486 nm/nM, optical transmittance sensitivities of 0.535504 × 10^−3^ 1/(ng/ml) and 18.91 × 10^−3^ 1/nM, total output sensitivities of 0.5398 (mA/W)/(ng/ml) and 19.059 (mA/W)/nM, and measurement ranges of 0-1000 ng/ml and 0-28.33 nM are achieved for PSA and Hepatitis DNA, respectively. Besides, a procedure of design for the measurement approach is provided.

## Electronic supplementary material

Below is the link to the electronic supplementary material.

Supplementary Material 1

## Data Availability

All data needed to evaluate the conclusions are presented in the paper. Furthermore, Mr. Hossein Bahramian can provide simulation files for whom with sensible reason (hossein7i7ba@gmail.com).
