# Supplementary figures and images for: Folded flexure MOEMS for the detection of PSA and hepatitis DNA as biosensor for prostate cancer and viruses
Source: Sci Rep. 2024 Oct 2;14:22881. doi: 10.1038/s41598-024-73910-x (PMC11446923; doi:10.1038/s41598-024-73910-x)

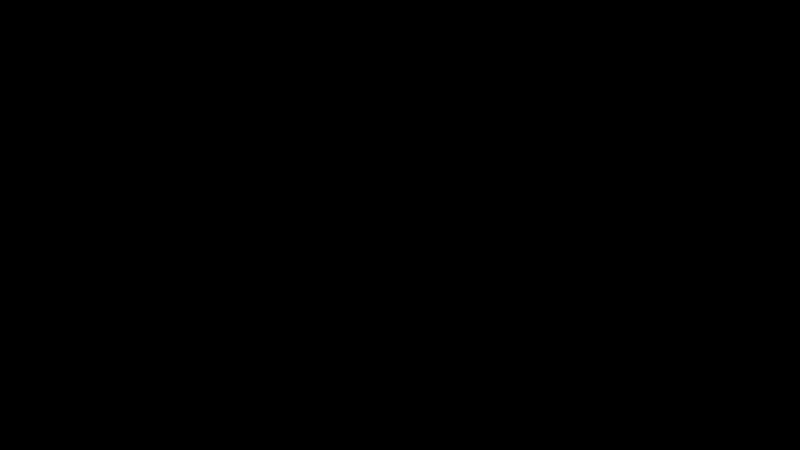

Supplement: Supplementary file 1 — Supplementary Material 1 [file 41598_2024_73910_MOESM1_ESM.gif]
